# Transcranial magnetic stimulation, synaptic plasticity and network oscillations

**DOI:** 10.1186/1743-0003-6-7

**Published:** 2009-03-02

**Authors:** Patricio T Huerta, Bruce T Volpe

**Affiliations:** 1 Weill Medical College at Cornell University, Department of Neurology and Neuroscience, Burke Cornell Medical Research Institute, 785 Mamaroneck Ave, White Plains, NY 10605, USA

## Abstract

Transcranial magnetic stimulation (TMS) has quickly progressed from a technical curiosity to a bona-fide tool for neurological research. The impetus has been due to the promising results obtained when using TMS to uncover neural processes in normal human subjects, as well as in the treatment of intractable neurological conditions, such as stroke, chronic depression and epilepsy. The basic principle of TMS is that most neuronal axons that fall within the volume of magnetic stimulation become electrically excited, trigger action potentials and release neurotransmitter into the postsynaptic neurons. What happens afterwards remains elusive, especially in the case of repeated stimulation. Here we discuss the likelihood that certain TMS protocols produce long-term changes in cortical synapses akin to long-term potentiation and long-term depression of synaptic transmission. Beyond the synaptic effects, TMS might have consequences on other neuronal processes, such as genetic and protein regulation, and circuit-level patterns, such as network oscillations. Furthermore, TMS might have non-neuronal effects, such as changes in blood flow, which are still poorly understood.

## Introduction

Transcranial magnetic stimulation (TMS) is a technique for studying brain function, with advantages that have become apparent to neuroscientists, neurologists, clinical psychologists and therapists. TMS is non-invasive, causes negligible discomfort to subjects, does not require anaesthesia, and can be applied with exquisite temporal precision by using the appropriate magnetic coils [1,2]. As a result, TMS has been embraced by an expanding community of researchers and has led to a surge of publications. The recent handbooks by Pascual-Leone *et al *[3], Walsh and Pascual-Leone [4], and Wasserman *et al *[5] are recommended for the interested parties.

TMS is an emergent technology and, as such, it has many hurdles to overcome [3-5]. Obvious limitations include the relatively low spatial resolution (~1 cm) and the inability to stimulate at high frequencies (over 50 pulses per sec). Another drawback is the rapid decay of the electric field from the source; a pulse given at the scalp's level reaches only ~2 cm in depth [5]. Therefore, TMS can readily activate superficial regions (such as cerebral cortex, cerebellum and spinal cord), but it cannot reach deeper brain regions (such as hippocampus, amygdala, striatum, thalamus and brainstem). It is foreseeable that technical improvements, such as novel magnetic coils with active cooling, deeper penetrating power and more focal spatial resolution, will help overcome the current restrictions. An inherent limitation of TMS, however, is the nonspecific nature of the neural activation that follows a pulse. The activated volume of brain tissue contains excitatory, inhibitory and neuromodulatory neuronal compartments, all with the potential of being concurrently stimulated. Therefore, caution should be exercised when interpreting TMS studies.

In this review, we discuss the neural mechanisms underlying TMS. This topic has not been studied as thoroughly as expected, probably because most investigators are still determining the full range of applications for this emergent technique [6]. It is widely accepted, however, that TMS involves a range of neuronal processes such as synaptic excitation, synaptic inhibition and synaptic plasticity [2,3,6-9]. Moreover, TMS seems to affect circuit-level patterns, such as network oscillations, as well as non-neuronal effects, such as changes in blood flow [10,11].

A detailed understanding of the neural mechanisms at work in TMS is highly desirable because of the steady rise in studies attempting to use TMS in therapeutic settings [12]. For instance, researchers have reasoned that TMS could help awaken dormant cortical areas in individuals who had recently suffered a stroke. However, it has taken several years of dedicated effort to implement stimulation protocols that produce reliable, albeit minor, beneficial effects [2,12-14].

## The effect of a single TMS pulse

**Figure 1:**
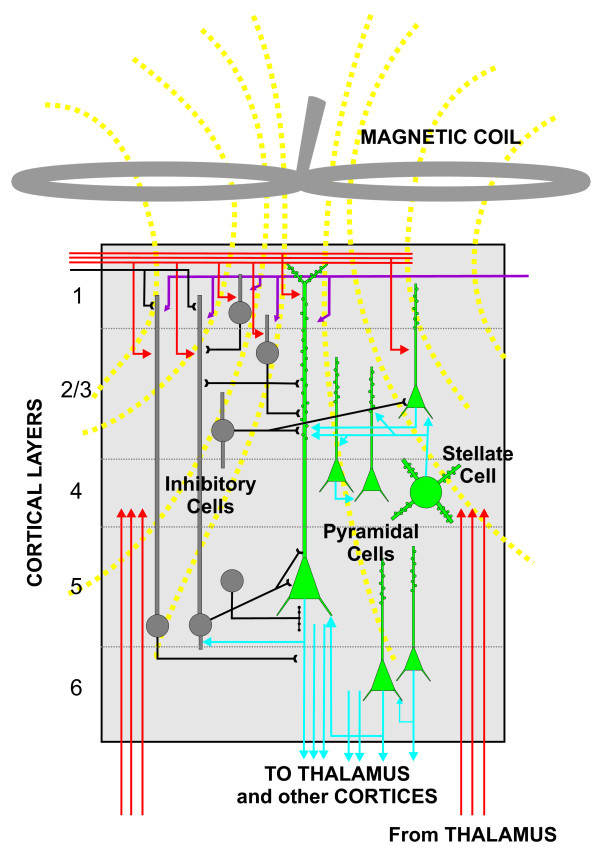
**Schematic representation of the human cerebral cortex**. The magnetic coil, represented as a figure-of-eight device, is placed on top of the cerebral cortex and pulses a magnetic field that induces electrical currents across the six layers of the cerebral cortex (indicated by numbers at left). The excitatory cells (green with blue axons) and the inhibitory cells (gray with black axons) have the potential to be activated at the level of their axons, which contain the highest density of ion channels. The incoming axons from other cortical areas and the thalamus (indicated in red) are also activated. The end result of the magnetic pulse is the synaptic activation of a chain of neurons, which generate feed-forward and feedback loops of excitation and inhibition.

In 1831, Faraday demonstrated that a rapidly changing magnetic field could induce an electrical current in a nearby conductor. In 1985 this principle was applied successfully to the cerebral cortex of the human brain [1]. This organ works as a conductor because the cells that reside within it maintain electrochemical gradients through a variety of ion channels and ion transporters. Therefore, when a single magnetic field is pulsed directly over the subject's head, via a specialized coil, it induces electrical currents across the different layers of the cerebral cortex (Fig. 1). A standard pulse lasts ~10^-5 ^sec and induces a magnetic field reaching up to 2 Tesla [2]. The magnitude of the pulse directly determines the volume of cortical tissue that is stimulated. Detailed simulations show that a 2 Tesla pulse activates a cylindrical volume (~1 cm radius, ~2 cm height), with an exponential decay from the central activation axis [5,15]. Because neuronal axons have the highest density of ion channels, they become preferentially activated during a weak magnetic pulse. When an axon becomes electrically active, an action potential travels along its axis until it reaches the presynaptic axon terminal. At this point, neurotransmitter is released onto the postsynaptic neuron. Most cortical neurons use the neurotransmitter glutamate and are classified as excitatory neurons. A smaller fraction of cortical neurons release γ-aminobutyric acid (GABA) and are classified as inhibitory neurons. Yet another group of neurons send long axonal projections from different brain nuclei to the cortex and release neuromodulators, such as acetylcholine, dopamine, norepinephrine, and serotonin. Therefore, even a weak TMS pulse always activates a mixture of excitatory and inhibitory neurons and has the potential to activate neuromodulatory pathways. Also, given the dense connectivity of cortical circuits, a TMS pulse potentially activates a chain of neurons, generating feed-forward and feedback loops of excitation and inhibition.

The behavioural response elicited by a single TMS pulse depends on the exact cortical area that is stimulated. When a pulse is given over the primary motor cortex (at the top of the head), it can induce twitches in the subject's muscles. In fact, a precisely localized magnetic pulse can lead to movement of a single finger. Similarly, a single pulse directed to the primary visual cortex (at the back of the head) can induce the sensation of seeing light, even when the eyes are closed, an experience known as a phosphene. In this sense, TMS is reminiscent of other techniques (such as electrical brain stimulation, positron emission tomography, and functional magnetic resonance imaging) that allow investigators to study specific cortical areas within dedicated sensory and motor modalities. Given the low spatial resolution of TMS, the technique does not allow for précised mapping of cortical areas.

The primary motor cortex (M1) constitutes the best-examined cortical region in terms of the effect of TMS [1-6]. One of the main reasons for this focused attention is the practical matter that even a weak, single TMS pulse applied over M1 can produce a muscle response, called a *motor evoked potential *(MEP), that is technically simple to measure. Indeed, the bulk of the TMS studies on M1 use the amplitude of the MEP as the single measure of TMS output. This potential is, however, separated by three synapses from the TMS source (1, synapses onto corticospinal neurons; 2, synapses onto motor neurons in the spinal cord and; 3, neuromuscular synapses). Nevertheless, careful studies have convincingly shown that a TMS pulse over M1 initiates a chain of events that begins with the stimulation of multiple axons distributed across the different cortical layers (Fig. 1). The axons of interneurons show the shortest latency to respond, which is followed by axonal activation of thalamo-cortical inputs and cortico-cortical inputs. The axonal activities of all these cells are synaptically integrated by the corticospinal pyramidal neurons in layer 5 and eventually lead to the generation of action potentials by the output cells (Fig. 1). These action potentials can be measured from the epidural space of the cervical spinal cord in conscious humans; they occur ~5–10 ms after the TMS pulse and have been termed *indirect waves *to emphasize the fact that they are the product of synaptic activation [2,5]. Once the corticospinal action potentials reach the spinal cord, they activate motor neurons. These cells in turn generate action potentials, which lead to the synaptic activation of muscles, ~20 ms after TMS. It is this activity that is measured as the MEP.

Interestingly, when a magnetic pulse is applied over a cortical area that is involved in cognition, it does not typically elicit an effect by itself. However, if the pulse is given when the person is involved in a cognitive task, it can greatly interfere with proper performance [3,15]. For instance, a single TMS pulse given over Broca's language area (located in the left hemisphere in most people) as the subject verbalizes can produce speech interference. Conversely, a single TMS pulse can have a facilitatory effect when it is applied shortly before a cognitive task. For example, a subject displays a shorter latency for naming an object when a single TMS pulse is given over Wernicke's language area 500–1000 ms before the subject is shown the object [16]. These results indicate that even a single TMS pulse can generate differential consequences depending on the activation state of the cerebral cortex at the moment of applying the pulse [17]. They also call attention to the importance of timing when TMS is used in respect to a particular external stimulus.

## Repetitive TMS and synaptic plasticity

TMS protocols that include multiple pulses are known as repetitive TMS. These protocols consist of precisely structured patterns that are characterized by the number of pulses, the frequency with which they are given, and the intensity of each stimulus. It has been determined that repetitive TMS engages a variety of neuronal mechanisms, besides axonal activation, as well as non-neuronal processes that might be collectively responsible for the range of observed effects [4,11].

Remarkably, some protocols of repetitive TMS can elicit residual effects that persist for many minutes. In a serendipitous manner, the TMS patterns that produce long-lasting changes tend to emulate, in the stimulation regimens at least, the patterns that trigger synaptic plasticity in the hippocampus. This suggests that, at minimum, repetitive TMS harnesses the neural processes responsible for triggering changes among synaptic connections in cortical networks. Therefore, we will briefly describe the principles of synaptic plasticity and local inhibition in the rodent hippocampus before scrutinizing to what extent repetitive TMS might engage cortical synaptic plasticity.

### Synaptic plasticity in the hippocampus

**Figure 2:**
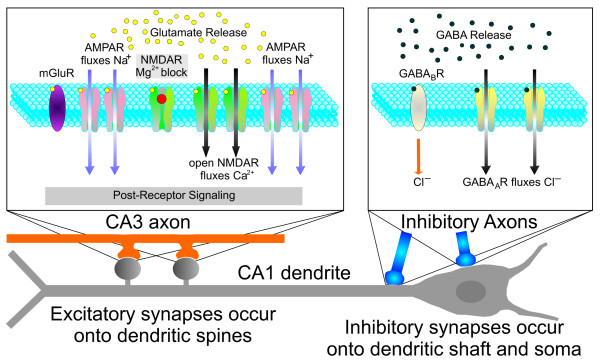
**Schematic representation of the glutamatergic and GABAergic receptors in a CA1 pyramidal neuron**. The left box represents a CA3-CA1 synapse. The CA3 axon (orange) releases glutamate from the presynaptic terminals. The postsynaptic CA1 neuron expresses three types of glutamatergic receptors: metabotropic receptor (mGluR), alpha-amino-3-hydroxy-5-methyl-4-isoxazolepropionic acid receptor (AMPAR), and N-methyl-D-aspartate receptor (NMDAR). The AMPARs are represented in their active state, as they allow Na^+ ^to enter onto the dendritic spine. The NMDARs are represented both in the closed state (leftmost NMDAR, with the Mg^2+ ^block seen as a red ball in the mouth of the receptor) and in the open state, when the NMDARs allow Ca^2+ ^to enter onto the spine (notice the absence of the Mg^2+ ^block). The right box represents a synapse between an inhibitory interneuron and the CA1 cell. The interneuron releases γ-aminobutyric acid (GABA) onto the CA1 pyramidal neuron, which expresses GABA_A _receptors (yellow) and GABA_B _receptors (gray), leading to inhibition of the target cell. The GABA_A _receptors are represented in the open state when they allow Cl^- ^to enter onto the CA1 dendrite.

From the wealth of information available [18,19], we will focus on the synaptic molecules and the patterns of electric stimulation that trigger synaptic plasticity in the CA1 region of the rodent hippocampus (Fig. 2). The excitatory synapses between the axons of CA3 neurons (the inputs) and the dendritic spines of CA1 pyramidal neurons (the targets) have been intensely studied [18,19]. The CA3 axon terminals release glutamate while the CA1 neurons express three types of glutamatergic receptors: alpha-amino-3-hydroxy-5-methyl-4-isoxazolepropionic acid receptor (AMPAR), N-methyl-D-aspartate receptor (NMDAR), and metabotropic receptor (mGluR). The AMPAR and the NMDAR function as ion channels that permeate positively charged ions when they are activated, depolarizing the neuron.

The strength of hippocampal synapses can increase dramatically following high frequency stimulation (HFS) of the inputs. Since the synaptic enhancement may persist for hours, or even days, it is called long-term potentiation or LTP [18,20,21]. There are several HFS patterns that can elicit LTP, but the most common consists of a single train of 100 Hz for 1 sec (100 pulses with 10-ms intervals). Another HFS protocol is called *theta burst stimulation *(TBS) and consists of 10 bursts (each burst is 4 pulses at 100 Hz) that are separated by an interval of 200 ms from each other [22]. The term theta refers to the fact that 200 ms is the main periodicity of the theta rhythm, a network oscillation that occurs during periods of heightened attention, such as when an animal explores a new environment [18]. Another HFS protocol is called *primed burst stimulation *[23], and consists of a single pulse that is followed by a burst (4 pulses at 100 Hz) with an interval of 200 ms. Indeed, even a non-primed burst can induce LTP by itself, if it occurs at the peak of a wave in theta rhythm [24]. This paradigm exploits the association of a network oscillation with a finely timed stimulating burst. Another associative protocol for LTP induction is called *spike-timing-dependent plasticity *[25-27]. It relies on the delivery of two pulses; the first triggers an action potential (or spike) in the input axon, while the second triggers an action potential in the target neuron. To elicit LTP, the input spike must precede the target spike (by 5–50 ms) and the pairing must occur many times. A typical protocol repeats the two pulses (input 10 ms before target) for 50 times at a frequency of 10 Hz [25].

The sequence of events underlying LTP induction is clearly understood [18,28]. When glutamate binds to the AMPAR, this receptor opens its pore for a brief period (10–20 ms), allowing Na^+ ^to enter into the dendritic spine, resulting in a small degree of depolarization. The NMDAR does not open immediately because its pore is blocked by Mg^2+ ^ions. HFS seems to be essential for removing the Mg^2+ ^block of the NMDAR, probably because HFS activates numerous AMPARs thus generating a large depolarization in the dendritic spine. When the NMDAR opens, it permeates Na^+ ^and Ca^2+ ^ions for hundreds of milliseconds. The resting Ca^2+ ^concentration in the cell's cytoplasm is very low (~10^-9 ^M) but when many NMDARs open during HFS, Ca^2+ ^reaches a high concentration (~10^-3 ^M) within the spine that activates several kinases, particularly calcium-calmodulin kinase II [29], and leads to phosphorylation and upregulation of the AMPAR.

The strength of hippocampal synapses can decrease persistently following low frequency stimulation (LFS), a process that has been termed long-term depression or LTD [30]. The most frequent LFS protocol is a single train of 1 Hz for 10 min (600 pulses) or for 15 min (900 pulses). Another effective protocol is paired pulse LFS [31,32] consisting of a train of paired pulses (2 pulses with a 200-ms interval) at 1 Hz for 15 min (1800 pulses). LTD can also be elicited by spike-timing-dependent plasticity [25-27], in which the target spike precedes the input spike (by 5–50 ms) and both spikes occur many times. Remarkably, this LTD induction protocol simply reverses the order of the target and the input spikes from the LTP induction protocol. Surprisingly, LTD induction also depends on the NMDAR. It is generally accepted that during LFS the NMDAR is mildly stimulated, producing an intermediate Ca^2+ ^elevation (~10^-6 ^M) that activates protein phosphatases and leads to dephosphorylation and down-regulation of the AMPAR [18,33].

### Inhibition in the hippocampus

The local interneurons in the CA1 region release GABA onto the CA1 pyramidal neurons, which express GABA_A _receptors and GABA_B _receptors, leading to inhibition of these target cells (Fig. 2) [34,35]. Since the CA3 axons have synaptic connections with the local interneurons, the activation of CA3 axons results in initial excitation of CA1 pyramidal cells (via the glutamatergic synapses) that is followed by feed-forward inhibition from the interneurons. Furthermore, the axons of CA1 pyramidal neurons themselves connect to the interneurons, so that when a CA1 pyramidal cell generates an action potential, it leads to rapid feedback inhibition. In this manner, the local interneurons are extremely effective in dampening excessive excitation of the CA1 pyramidal cells through the activation of feed-forward and feedback inhibitory loops.

Notably, the local interneurons express GABA_B _autoreceptors in their presynaptic terminals that stop the release of GABA after ~200 ms [18,36]. This fact explains the tremendous efficacy of TBS and primed burst stimulation for inducing LTP, as well as paired pulse LFS for inducing LTD. In each of these protocols, one of the consequences of the first pulse is to trigger GABA release from the interneuronal terminals, which then blocks its own release at the exact time (200 ms) that the second stimulus occurs. If the second stimulus is a single pulse, it triggers mild NMDAR activation that leads to LTD. If the second stimulus is a burst of pulses, it elicits strong NMDAR activation and subsequent LTP.

### Lessons from the hippocampus applied to repetitive TMS

Many reports have demonstrated that the principles of synaptic plasticity that were first uncovered in the hippocampus can be extended to the cerebral cortex [37-46]. In particular, NMDARs and AMPARs seem to play similar roles in the long-term plasticity of cortical synapses as they do in the hippocampus [37]. Moreover, the local interneurons in the cerebral cortex exert strong inhibitory influences over the pyramidal and stellate neurons [47]. However, a crucial difference between these brain regions is that the cortical networks are structurally much more complex than the hippocampal circuits. Cortical neurons are placed in multilayered arrangements (the canonical six layers), with copious synaptic connections within each functional module and with numerous axons running from each module to its connected counterparts (Fig. 1). Also, cortical neurons receive massive inputs from the thalamus and, in turn, project heavily to the same structure. Therefore, there are vast recursive loops of excitation and inhibition between the cortex and the thalamus, as well as between the different areas of the cortex, including loops between both cerebral hemispheres.

Given the structural complexity of the cerebral cortex, it might be surprising that TMS protocols that emulate the induction paradigms for LTP and LTD (in rodents) would be successful in modifying the efficacy of cortical networks in humans. A parsimonious explanation is that patterned TMS can trigger changes in the human cortical synapses that are similar, at the mechanistic level, to the plasticity that occurs in rodent cortical synapses when they undergo LTP or LTD. Although this is a tentative proposal, it is supported by the observation that the most effective TMS protocols (for producing long-term change) mirror closely the protocols used for inducing LTP and LTD in rodent preparations. Two straightforward predictions of this conjecture are: (i) minor deviations from the prescribed LTP and LTD induction protocols would be much less efficient in producing TMS-induced plasticity, (ii) pharmacological agents that block LTP and LTD induction in rodents would be effective in blocking the TMS-induced plasticity.

Thus far, M1 has been the most investigated cortical region with regards to TMS-induced plasticity [2,6,15]. The current evidence highlights the critical effectiveness of TMS protocols that mimic the induction paradigms for LTD and LTP. These TMS protocols invariably produce changes in MEP amplitude that outlast the TMS application [5,12]. It must be noted, however, that using the MEP as the sole readout of TMS-induced plasticity is problematic because the MEP is removed by three synapses from the source of TMS (as detailed above), whereas LTP and LTD are monosynaptic events. It would thus be highly desirable to monitor a cortical readout that is linked by a single synapse to the TMS pulse. Studies in which TMS is coupled with recording techniques such as high-density electroencephalography have the potential to provide such direct monosynaptic readout.

When a train of TMS pulses is applied at 1 Hz, it leads to lasting decrease of the MEP [5,48-51]. In one of the original reports, Chen *et al *[48] showed that repetitive TMS at 0.9 Hz applied for 15 min (810 pulses), with a stimulation intensity set at 115% of the resting motor threshold, produced 20% decrease of the MEP that lasted for ~15 min. Touge *et al *[49] used repetitive TMS at 1 Hz, with an intensity of 95% of resting threshold, applied for 25 min (1500 pulses) and obtained a 50% decrease of the MEP that returned to the pre-TMS baseline in ~30 min. Thus, the application of a longer 1-Hz train was able to induce a stronger depression that persisted for a somewhat longer period. These results are in line with the LTD studies in rodents.

It has been shown that high frequency patterns of TMS given over M1 can increase cortical efficacy. In a pioneer study, Pascual-Leone *et al *[52] used a train of 10 pulses of TMS at 20 Hz, with an intensity of 150% of resting threshold, and obtained a 50% increase of the MEP that lasted for ~5 min. This result is reminiscent of the rodent studies in which an induction protocol of intermediate frequency (i.e., 20 Hz) produces a transient synaptic enhancement that is called short-term potentiation.

Unfortunately, overheating of the magnetic coils prevents investigators from using the classical protocol for inducing LTP (100 Hz for 1 sec). Moreover, there is a nontrivial possibility that such high frequency stimulation may lead to seizures in susceptible individuals. Given these caveats, some studies have used trains of lower frequency in an attempt to enhance efficacy. For example, modest increases of the MEP are obtained following TMS trains at 5 Hz [53,54]. It is important to realize that in rodent studies of synaptic plasticity, a 5-Hz protocol does not fall within the frequency range that would induce LTP. If anything, it might be easier to induce LTD because single pulses at 5 Hz are very effective in mildly activating NMDAR and in suppressing GABA release (through activation of the GABA_B _auto-receptors). In fact, the landmark study by Allen *et al *[55] in the cat primary visual cortex clearly demonstrated that TMS trains of 1–8 Hz for 1–4 sec were all capable of depressing visually evoked responses, which were quantified as the rate of action potentials of the cortical neurons that were triggered by a visual stimulus. For example, following a brief TMS train of 4 Hz for 2 sec (8 pulses), the rate of action potentials was greatly depressed for more than 5 min. A visual stimulus that before TMS produced ~80 action potentials per sec was unable to trigger a single event during the initial 2 min post-TMS. The cortical activity slowly recovered to 40 action potentials per sec in response to the visual stimulus 5 min after TMS.

An exciting development in the search for TMS protocols that enhance cortical efficacy has occurred recently. Several investigators have demonstrated that the TBS protocol used for LTP induction can produce a lasting increase in cortical activity [56-59]. Huang *et al *[56] measured a 50% increase in the MEP, that lasted ~20 min, following a protocol they called intermittent TBS. Their protocol consisted of 600 pulses, with an intensity of 80% of resting threshold, that were distributed in 20 episodes according to the following scheme: each episode consisted of a burst of three TMS pulses (at 50 Hz, 20 ms between each pulse) that was repeated at 5 Hz for 2 sec (for a total of 10 bursts). A silent interval of 8 sec followed and then a new episode was applied. Interestingly, when the 50-Hz bursts were applied in a continuous fashion (that is, the bursts were repeated at 5 Hz with no intervening silent period), the MEP was depressed. Esser *et al *[57] combined an intermittent TBS protocol with high-density electroencephalographic measurements and found that intermittent TBS over M1 in the left hemisphere enhanced the MEP in the right hand, as expected, but it also increased neural responses in the premotor cortex bilaterally. Therefore, the intermittent TBS protocol was not only able to affect the motor output, but also the efficacy of cortical areas closely related to M1.

The question of whether the post-TBS enhancement displays the NMDAR dependence that would be expected of an LTP mechanism has been recently addressed with the use of the NMDAR antagonists memantine (uncompetitive antagonist) and D-cycloserine (competitive antagonist at high doses) [60,61]. A small amount of memantine (4 doses of 5 mg each, over 2 days) given before TMS, can completely block the facilitatory effect of intermittent TBS and, also, the suppressive effect of continuous TBS [61]. Critically, memantine blocks training-induced motor cortex plasticity, does not commonly produce side effects, and has good blood-brain barrier penetrating rate [62-66]. A dose of D-cycloserine (100 mg, taken 2 hours before TMS) can turn the facilitatory effect of intermittent TBS into a depressive effect [62]. These results are encouraging and, together with the bulk of the TMS studies tend to support the conjecture that synaptic plasticity might mediate the long-term changes in cortical efficacy generated by TMS protocols that mimic LTP and LTD induction paradigms.

Recent studies have explored associative protocols in which TMS is combined with peripheral nerve stimulation to generate plasticity [67-71]. It has been proposed that these protocols follow the association principles of spike-timing-dependent plasticity. For instance, the pioneer study by Stefan *et al *[67] delivered an electrical stimulus to the right median nerve in the wrist that was followed (25 ms later) by a TMS pulse over the left hemisphere at the optimal site for activating the abductor pollicis brevis muscle. This paired stimulation was repeated 90 times, with an interval of 20 sec, and produced a 55% increase in MEP amplitude that returned to baseline in ~1 hour. To explain this result in terms of spike-timing-dependent plasticity, one needs to argue that the medial nerve stimulation provides the presynaptic spike, whereas the TMS pulse provides a precisely timed postsynaptic spike. Indeed, medial nerve stimulation triggers an action potential that takes ~20 ms to travel from the wrist to the somatosensory cortex and ~3 ms for propagating from the somatosensory cortex to M1. This means that the TMS pulse (given 25 ms after medial nerve stimulation) occurs ~2 ms after the input arriving from the somatosensory cortex. It is therefore possible that the presynaptic spike and the postsynaptic spike occur with the precise timing required for LTP. Although other conceptual scenarios might be able to explain the results obtained with the associative protocols, they could feasibly represent a genuine realization of the principles of spike-timing-dependent plasticity in the human cortex.

## TMS and network oscillations

The analysis of how TMS might influence circuit-level events, such as network oscillations, constitutes an emerging area of research. A vast body of work has shown that cortical oscillations represent a signature of ongoing operations occurring in intrinsic cortico-cortical loops and cortico-thalamic circuits [72]. At every moment in time, there is a discrete ensemble of cortical neurons that is active and, when this ensemble becomes silent, it is instantly replaced by a new set of active neurons. This constant wave of neural activation and silencing all over the cortical mantle gives rise to short-lived oscillations that wax and wane according to the brain's internal dynamics [73]. Notably, the cortical ensembles generate oscillatory bands that cover an enormous range of frequencies (0.02 Hz to 600 Hz). In the waking brain, when attending to external stimuli, many cortical ensembles synchronize in the gamma frequency range (30–80 Hz). Therefore, it has been suggested that gamma oscillations reflect the *binding *(putting together) of the features of external stimuli [72,74]. In the absence of sensory inputs, the most prominent oscillations in the waking brain are in the alpha range (8–12 Hz), and it is thought that alpha oscillations reflect partial disengagement from the environment or internal mental processing [72]. During deep sleep, several slow waves occur, such as the slow 1 oscillation (0.5–0.7 Hz) and the delta oscillation (1.5–4 Hz). It has been suggested that these sleep waves are involved in the process of memory consolidation, although the exact mechanisms have not been identified [75].

Recent TMS studies have measured the consequences of TMS on network oscillations, with the use of concomitant high-density electroencephalography [76-82]. For example, Massimini *et al *[76] have found that, during quiet wakefulness, a TMS pulse over the premotor cortex (in the right hemisphere) induces a sequence of time-locked gamma oscillations (20–35 Hz) in the first 100 ms, followed by a few slower (8–12 Hz) components that persist until 300 ms. These travelling waves propagate to connected cortical areas, even several centimetres away. Remarkably, during deep sleep, the response to the TMS pulse is radically different, consisting of a single wave of high amplitude in the premotor site that lasts for ~200 ms and does not propagate to the connected areas. In another study, Massimini *et al *[81] have shown that a TMS pulse over the sensorimotor cortex can trigger a high-amplitude slow wave during sleep that spreads over the whole cortical mantle, and it is reminiscent of the naturally occurring slow oscillation. Since this type of oscillation has been postulated to play a role in memory consolidation, this study opens the possibility of examining this elusive process with TMS technology.

The work by Allen *et al *[55] in the cat visual cortex represents the most throughout mechanistic study of multiple effects of TMS. The authors measure robust decreases in action potentials, but they also investigate the consequences of TMS on the local network oscillations and the local blood flow. Immediately after TMS, the spontaneous local field oscillations show a great increase in the high frequency band (oscillations between 50–150 Hz) that lasts for ~60 sec. This is consistent with the idea that inhibitory loops are recruited. Moreover, the spontaneous local field oscillations in the lower band (<40 Hz) show a sustained reduction, suggesting an effect on the oscillatory processes that participate in sensory binding. In a technical tour de force, Allen *et al *[55] also report the levels of tissue oxygen in the visual cortex and find that oxygen is well correlated with the occurrence of action potentials. In fact, the lowest levels of oxygen are recorded after the 8-Hz protocol that also elicits the strongest decrease in the number of action potentials in response to a visual stimulus.

Current encephalographic analysis is a robust methodology with multiple applications in basic and clinical neuroscience. It is expected that the studies that combine high-density electroencephalography with TMS will continue to illuminate the role of network oscillations in the cerebral cortex, as they represent unique markers of neural processes such as sensory binding, memory consolidation and mental ideation. TMS can easily add the much-needed predictive component to these investigations [82].

## Other effects of TMS

TMS seems to have several consequences that are not directly related to synaptic plasticity and neuronal excitability. Such effects are just starting to be examined experimentally. The results thus far suggest that repetitive TMS protocols can trigger the activation of neuromodulators, such as acetylcholine, dopamine, norepinephrine and serotonin [83-89]. Presumably, these substances would be released during the TMS protocols and would continue to exert their modulatory effects after TMS has terminated. In fact, neuromodulators are constantly released onto the cerebral cortex in coordination with certain behavioural states. It would be expected that weak TMS protocols, such as single-pulse TMS, would have only minor influences over the ongoing release of neuromodulators. Conversely, patterned TMS paradigms (lasting for several minutes) would be expected to facilitate the release of at least some neuromodulators. Preliminary experiments in rats tend to agree with this premise [83,84], but much work remains to be done. Recently, it has been shown that TMS can trigger the expression of brain-derived neurotrophic factor and plasticity-related genes [90-92]. Moreover, TMS could help in phenotyping individuals with genetic mutations that affect cortical excitability, such as a mutation affecting the gene encoding the GABA_A _receptor [93], serotonergic gene polymorphisms [94], and the D90A superoxide dismutase-1 gene mutation [95].

TMS has already been incorporated to the arsenal of therapeutic tools that are used to mitigate the negative effects of neurological conditions, but these novel results open the exciting possibility that TMS also becomes a tool for manipulating the release and expression of endogenous trophic factors and beneficial gene products. This topic needs to be investigated further, but its high relevance makes it an attractive research focus for clinical researchers.

## Conclusion

We have discussed the biological mechanisms that are most likely to be engaged when TMS is applied over the cerebral cortex. It is clear that TMS can activate a host of neural phenomena, at different levels of organization, from synaptic plasticity to circuit-level oscillations. We have proposed that only a handful of the TMS protocols that are currently used for producing changes in cortical efficacy have the credentials for generating synaptic plasticity, similar to LTP and LTD. We have also mentioned that TMS may influence a large variety of non-neuronal processes that have yet to be fully elucidated.

## Competing interests

The authors declare that they have no competing interests.
